# Alginate–Gelatin Self-Healing Hydrogel Produced via Static–Dynamic Crosslinking

**DOI:** 10.3390/molecules28062851

**Published:** 2023-03-22

**Authors:** Francesca Cadamuro, Valeria Ardenti, Francesco Nicotra, Laura Russo

**Affiliations:** 1 Department of Biotechnology and Biosciences, University of Milano-Bicocca, Piazza della Scienza 2, 20126 Milano, Italy; 2 CÚRAM, SFI Research Centre for Medical Devices, National University of Ireland Galway, H91 W2TY Galway, Ireland

**Keywords:** static–dynamic, hydrogel, ECM mimetic, alginate, gelatin

## Abstract

Alginate–gelatin hydrogels mimicking extracellular matrix (ECM) of soft tissues have been generated by static–dynamic double crosslinking, allowing fine control over the physical and chemical properties. Dynamic crosslinking provides self-healing and injectability attributes to the hydrogel and promotes cell migration and proliferation, while the static network improves stability. The static crosslinking was performed by enzymatic coupling of the tyrosine residues of gelatin with tyramine residues inserted in the alginate backbone, catalyzed by horseradish peroxidase (HRP). The dynamic crosslinking was obtained by functionalizing alginate with 3-aminophenylboronic acid which generates a reversible bond with the vicinal hydroxyl groups of the alginate chains. Varying the ratio of alginate and gelatin, hydrogels with different properties were obtained, and the most suitable for 3D soft tissue model development with a 2.5:1 alginate:gelatin molar ratio was selected. The selected hydrogel was characterized with a swelling test, rheology test, self-healing test and by cytotoxicity, and the formulation resulted in transparent, reproducible, varying biomaterial batch, with a fast gelation time and cell biocompatibility. It is able to modulate the loss of the inner structure stability for a longer time with respect to the formulation made with only covalent enzymatic crosslinking, and shows self-healing properties.

## 1. Introduction

The mechanical properties of extracellular matrices (ECMs) exploit a fundamental role in cell functions and fate regulation. Biomolecular identity of ECM components is strongly involved in cell fate induction and regulation in both physiological tissue development and maintenance and in the induction of pathological states [1,2]. ECM stiffness and viscoelastic properties are also important in cell–matrix signaling, and are associated to many cellular pathological processes [2,3,4].

The generation of crosslinked hydrogels, taking advantage of covalent crosslinking strategies, allows the production of biomaterial platforms with fixed chemical and physical features useful to study cell fate modulation in tissue regeneration. These hydrogels can be remodeled by the cells embedded during construct maturation. However, cells’ microenvironment is characterized by static interactions that maintain the architecture and the structure of tissues and dynamic interactions indispensable for cell fate control and signal transduction. The introduction of reversible and dynamic crosslinking offers the unique opportunity to recreate and mimic the dynamic nature of ECM, recreating a complex and multifunctional network in which cells can adhere, differentiate, and proliferate, recreating the adequate tissue microenvironment.

Viscoelastic hydrogels can be generated by exploiting reversible crosslinking [3,5,6], and to this purpose different classes of dynamic covalent bonds have been explored, including imines [3], disulfides [7,8] Diels–Alder adducts [9,10], and boronic esters [11,12].

Boronate–diol interactions, in particular, emerged as a promising model for tissue engineering and injectable materials, showing self-healing properties, pH- and glucose-responsive behaviors and dynamic rheological properties [11]. Boronate ester formation, however, is favored at pH values above physiological pH [11] since the pKa of phenylboronic acid is 8.8 [13]. This limits the stability of the hydrogel, compromising the gelation and limiting long cell culture experiments [14]. A possible solution to overcome this problem consists in a careful dosage of a second crosslinking [15], this time with irreversible covalent bonds, to give mechanical support to the final “dual crosslinked” hydrogel [15,16,17,18,19]. However, despite some examples of static–dynamic hydrogels made only by polysaccharides or synthetic polymers [20,21,22], in the literature there is a lack of hybrid formulations with a protein and polysaccharide components, able to mimic the natural tissues.

In this work, we describe the generation of static–dynamic hydrogels exploiting alginate, largely employed as a hydrophilic hydrogel component [23,24,25], and gelatin that contains the RGD sequence for cell adhesion and guarantees the mechanical support to the final matrix. In order to obtain the static crosslinking, enzymatic crosslinking catalyzed by horseradish peroxidase (HRP) was performed by taking advantage of tyramine residues inserted in the alginate backbone and tyrosine residues in gelatin. To introduce the dynamic crosslinking, alginate was functionalized with 3-aminophenylboronic acid to obtain reversible bonds between the vicinal hydroxyl groups of the alginate chains and residual carbohydrate content of gelatin.

## 2. Results and Discussion

**Scheme 1:**
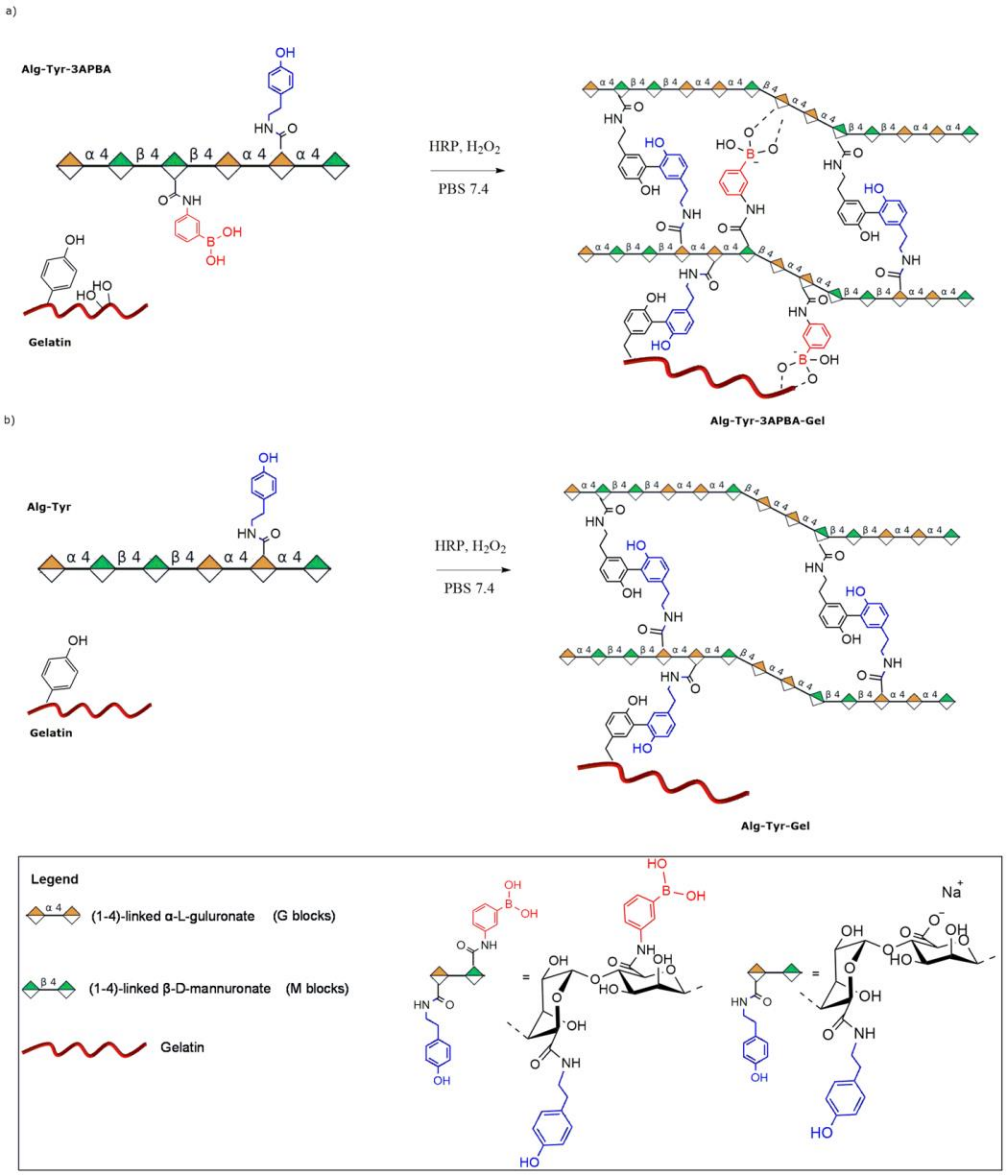
(**a**) Static and dynamic crosslinking generating the Alg-Tyr-3APBA-Gel hydrogel. Tyramine and 3APBA randomly attach to the carboxylic acid group of mannuronic or guluronic residues (respectively, green and orange diamonds represent D-mannuronic and L-guluronic residues). (**b**) Static Alg-Tyr-Gel hydrogels.

The strategy to generate static–dynamic 3D hydrogels is based on the double crosslinking of alginate and gelatin, combining and dosing reversible and irreversible covalent bonds. As an additional advantage, the proposed method allows obtaining hybrid hydrogels to perform a double functionalization using a single step, without the need for further functionalization of the gelatin partner usually needed in the generation of the final desired hydrogel. Alginate contains 1,2-diol sequences suitable for a dynamic crosslinking with boronic acid residues, once inserted in the polymeric chains. For static crosslinking, we decided to engage the few tyrosine residues (<1%) of gelatin, exploiting the horseradish peroxidase (HRP)-catalyzed [26] enzymatic coupling with tyramine residues inserted in the alginate backbone (Scheme 1). In detail, tyramine and 3-aminophenylboronic acid (3APBA) randomly attach to the carboxylic acid group of mannuronic or guluronic residues of alginate, allowing the static–dynamic linkages between alginate and gelatin (Scheme 1a). The covalent and static crosslinking between alginate and gelatin is ensured by HRP-catalyzed crosslinking (Scheme 1b).

### 2.1. Alginate Functionalization

Alginate was the only hydrogel component that needed functionalization. It was double functionalized in one step with tyramine [26] and 3-aminophenylboronic acid (3APBA) [27], requiring only one final purification step. To this purpose, the carboxylic acid groups of alginate were activated with N-(3-dimethylaminopropyl)-N’-ethylcarbodiimide hydrochloride (EDC) and N-hydroxysuccinimide (NHS) to react with the amine group present in both 3APBA and tyramine. It is noteworthy that not all the carboxylic acid groups of alginate must be converted into amides, being fundamental to guarantee the physical properties and solubility of the polymer. Experimentally, the addition of tyramine was dosed to reach a degree of functionalization of about 10% [26,28], and the subsequent addition of 3APBA reached a degree of functionalization of about 6%.

#### Characterization of Alginate Functionalization

Alginate and functionalized alginate were characterized by ^1^H and ^13^C nuclear magnetic resonance (NMR) and Fourier-transform infrared spectroscopy (FT-IR).

^1^H-NMR. The degree of functionalization (DoF) of alginate was determined by ^1^H NMR. The spectrum of Alg-Tyr-3APBA was compared with that of sodium alginate (the spectra of tyramine [29] and 3APBA [12] are reported in SI Figure S1). The signals of the aromatic protons of 3APBA partially overlapped the signals of two of the aromatic protons of tyramine in the range of δ = 6.8–7.7 ppm; peaks of 3APBA were identified in a range of δ = 7.3–7.7 ppm [23] while the aromatic protons in the *orto* position with respect to the hydroxyl group peaks of tyramine were visible at δ = 6.8. The protons in the meta position of tyramine were visible at δ = 7.2 ppm [30].

**Figure 1:**
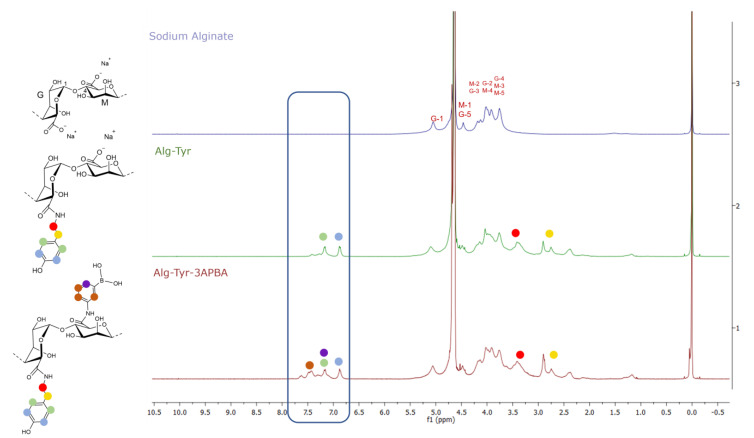
^1^H-NMR of sodium alginate (blue), Alg-Tyr (green), and Alg-Tyr-3APBA (red). ^1^H-NMR of tyramine and 3APBA is reported in SI. The functionalization of alginate was confirmed by the presence of protons of tyramine, which are visible in the range of δ = 6.8 to δ = 7.2 ppm, while peaks of 3APBA were identified in a range of δ = 7.3–7.7 ppm, partially overlapped with tyramine peaks.

The degree of functionalization of Alg-Tyr-3APBA was 6% for tyramine and 10% for 3APBA. ^13^C-NMR analysis was performed to further verify the effective functionalization of the sodium alginate (SI Figure S2). As reported in SI Figure S2, in ^13^C-NMR analysis of Alg-Tyr-3APBA, a peak at δ = 174 ppm appears, related to the formation of the amide group which is, as expected, totally absent in sodium alginate spectra [31]. The peak at δ = 175 ppm is instead related to the carboxyl group, and it is present in both compounds. This means that the polymer is not completely functionalized which agrees with the relatively low DOF% values calculated from ^1^H-NMR. Moreover, in Alg-Tyr-3APBA are visible peaks at δ = 130 ppm and at δ = 115 ppm which are related to the *orto* and *meta* carbon of the aromatic ring of tyramine adduct [32]. In the range δ = 128–135 ppm are visible the peaks related to the carbon atoms of aromatic ring of phenyl boronic acid adduct [27]. Figure 1 also reports the spectrum of alginate functionalized only with tyramine. Aromatic protons of tyramine were visible in the range of δ = 6.8–7.2 ppm [30] and the degree of functionalization was 5.5%.

FT-IR. Sodium alginate, Alg-Tyr, and Alg-Tyr-3APBA were investigated with FT-IR analysis. In Figure S3 (SI) the spectrum of sodium alginate is reported, in which a broad band centered at 3300 cm^−1^ is observed, related to hydrogen bonded [O−H] stretching vibration; at 2927 cm^−1^ a weak band of the stretching vibration of aliphatic [C–H] bond; at 1602 cm^−1^ asymmetric stretching vibration and at 1410 cm^−1^ symmetric stretching of carboxylate [C=O] of the carboxylic group of alginate. Then, the sharp peak at 1023 cm^−1^ may be also be attributed to [C−O] stretching vibration. The other peaks that appear in the region between 1190 cm^−1^ and 900 cm^−1^ are also characteristic of the sodium alginate polysaccharide structure and related to [C–O] and [C–C] stretching vibrations [33,34]. The peaks at 1517 cm^−1^ is associated with C-C stretching vibration of the aromatic ring of tyramine, present in spectra of both Alg-Tyr-3APBA and Alg-Tyr, while it is absent in the sodium alginate control spectrum. Confirmation of the functionalization of Alg-Tyr-3APBA and Alg-Tyr in comparison to the control spectrum involved the modifications of peaks at 948 cm^−1^ and 902 cm^−1^, which are attributed to the C-O stretching of uronic acid residues and C-H deformation vibration of β-mannuronic acid residues [33], and the modification of the peak at 1316 cm^−1^ is attributed to the O-C-H stretching vibration of the pyranose ring [33].

### 2.2. Hydrogel Formulation

The double functionalized alginate (Alg-Tyr-3APBA) was crosslinked with gelatin to obtain Alg-Tyr-3APBA-Gel hydrogels (Scheme 1a). The dynamic crosslinking was based on the reversible boronic ester formation between the 3APBA inserted in the alginate chains and the many vicinal diols also present in surrounding alginate chains. The static crosslinking exploited the stable covalent bond between the tyramine residues inserted in the alginate chain and the tyrosine residues of gelatin, catalyzed by HRP activated by H₂O₂. The contribution of the static bond and the dynamic equilibrium at pH 7.4 had to be balanced in order to favor cell spreading and migration inside the model [14]. A formulation study was performed to identify the best compromise in terms of gelation time, stability, stiffness, viscosity, and mechanical properties of the final hybrid hydrogel. In order to investigate the contribution of dynamic interactions, static–dynamic hydrogel was compared to static hydrogel based on the covalent enzymatic crosslinking between tyramine-functionalized alginate (Alg-Tyr) and gelatin to obtain Alg-Tyr-Gel hydrogel (Scheme 1b). First, three different formulations were compared keeping the concentration of enzyme and hydrogen peroxide constant and varying the biomaterial quantities reported in Table 1.

Formulations Alg-Tyr-3APBA-Gel-a and Alg-Tyr-3APBA-Gel-b gelled in 1 min and 5 min, respectively, while Alg-Tyr-3APBA-Gel-c, presenting a lower concentration of tyramine, was unable to form a hydrogel. Tyramine:tyrosine ratios of 2.5:1 and 1:1 were selected for the following formulation study in which lower concentrations of HRP and H_2_O_2_ were used in order to minimize the potential cytotoxic effects of H_2_O_2_ (Table 1).

Both Alg-Tyr-3APBA-Gel-d and Alg-Tyr-3APBA-Gel-e were able to form a hydrogel, even if longer gelation times were necessary, of 5 and 15 min, respectively. Alg-Tyr-3APBA-Gel-e was discarded as the gelation time was too long, whereas Alg-Tyr-3APBA-Gel-d was the hydrogel of choice for subsequent experiments. To investigate the contribution of dual crosslinking to the hydrogel properties, Alg-Tyr-3APBA-Gel-d was compared with Alg-Tyr-Gel, obtained in the same experimental conditions (Table 1) but containing only covalent crosslinking. Both the hydrogels were analyzed and compared by swelling test, rheological analysis, self-healing test, and preliminary biological test.

#### 2.2.1. Swelling Test

The swelling properties of hydrogels influence cell distribution and proliferation and diffusion of nutrients and play a fundamental role in drug loading and releasing efficiency in hydrogels with drug delivery applications [35,36].

**Figure 2:**
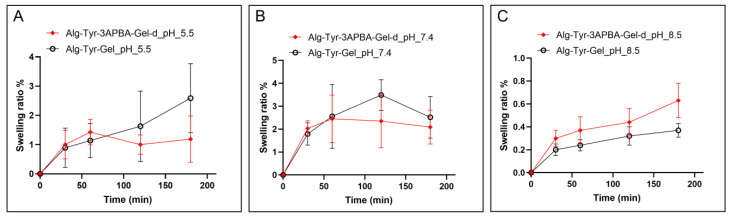
Swelling ratio % of Alg-Tyr-3APBA-Gel-d (red) and Alg-Tyr-Gel (black) at (**A**) pH 5.5, (**B**) pH 7.4, and (**C**) pH 8.5.

The swelling ratio of both static and static–dynamic hydrogels was evaluated and compared at pH 5.5, 7.4, and 8.5 and reported in Figure 2.

As expected, the swelling ratio decreases with the increase in pH from 5.5 to 8.5 due to the contribution of the alginate functional groups. However, slight differences in swelling trends are observable at pH 7.4 and pH 8.5 due to the contribution of boronate–diol interaction linkages that stabilize the hydrogels, showing comparable behavior in both static and static–dynamic conditions. Furthermore, the weight did not change significantly as reported in the literature for similar hydrogels [26,37,38].

#### 2.2.2. Rheological Characterization

**Figure 3:**
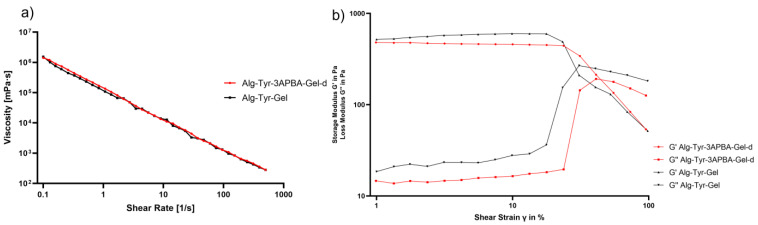
(**a**) Viscosity curves of Alg-Tyr-3APBA-Gel-d (red) and Alg-Tyr-Gel (black). Analysis was carried out at 37 °C in a range of shear rate of 0.1–500 s^−1^; (**b**) amplitude sweep analysis of Alg-Tyr-3APBA-Gel-d and Alg-Tyr-Gel.

Preliminary rheological characterization was performed on hydrogels Alg-Tyr-3APBA-Gel-d and Alg-Tyr-Gel. Figure 3 reports the viscosity curve obtained by plotting hydrogels’ viscosity versus shear rate. The viscosity curve of the two hydrogels was comparable and showed a non-Newtonian shear thinning behavior since the viscosity decreases as the shear rate increases. These properties are important for applications in tissue engineering and drug delivery systems [39].

An amplitude sweep experiment (Figure 3b) was performed to determine the linear viscoelastic region (LVER), which is the region where the applied stresses are insufficient to cause breakdown of the structure and end with the yield point. The results for both hydrogels showed G′/G″ >> 1, typical of stronger hydrogel. Hydrogel Alg-Tyr-3APBA-Gel-d presented a G′ constant value of 481 Pa ±5% in a shear strain range of 0.1% to 13%, while hydrogel Alg-Tyr-Gel showed the LVER of 0.1% to 23% with a constant value of 492 Pa ±5%. G′ value in LVER represents the stiffness of a hydrogel, therefore Alg-Tyr-Gel was stiffer than Alg-Tyr-3APBA-Gel-d. Moreover, the two hydrogels presented a different flow point, which is the value of shear stress at the crossover point (G′ = G″) and in which the viscous portion becomes dominant, and the sample starts to flow. Alg-Tyr-3APBA-Gel-d presented a flow point in the range of 41–55% shear strain, while Alg-Tyr-Gel at around 30% shear strain. It is possible to hypothesize that the 3APBA–diol interaction modulated the stiffness of the hybrid material and compensated for a longer time the loss of elasticity due to the shear stress, reaching the flow point later than the Alg-Tyr-Gel hydrogel.

#### 2.2.3. Self-Healing Test

**Figure 4:**
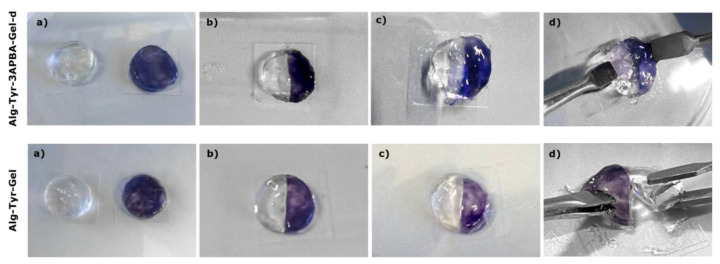
Self-healing test on hydrogels Alg-Tyr-3APBA-Gel-d (top) and Alg-Tyr-Gel (bottom). (**a**) Hydrogels after 30 min at 37 °C. (**b**) t₀—hydrogels just cut and manually reattached. (**c**) Hydrogels after 10 min at 37 °C. (**d**) Application of a tensile stress on hydrogels to test self-healing.

The self-repair property of a material damaged by external stimuli can be achieved through non-covalent or reversible dynamic bonds [40]. Self-healing tests were performed on hydrogels Alg-Tyr-3APBA-Gel-d and Alg-Tyr-Gel to investigate the contribution of the the dynamic boronic ester bond in the dual crosslinked hydrogel. As expected, the dual crosslinked hydrogel maintained a cohesive structure after the application of a tensile stress in the area where it was previously cut, while Alg-Tyr-Gel remained broken (Figure 4).

#### 2.2.4. Cytotoxicity

**Figure 5:**
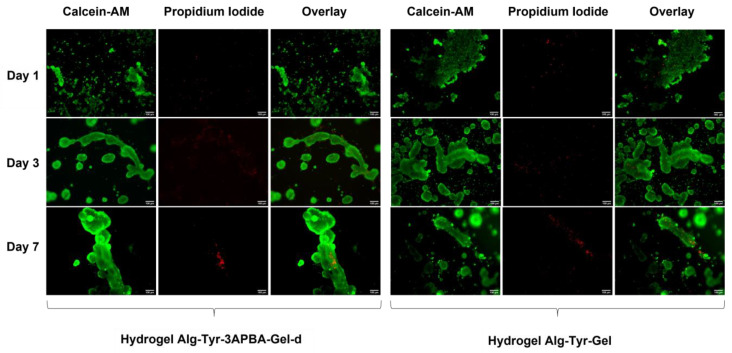
HT29 fluorescence images of live/dead assay at day 1, day 3, and day 7 on hydrogels Alg-Tyr-3APBA-Gel-d (**left**) and Alg-Tyr-Gel (**right**). Scale bar: 100 µm.

The cytotoxicity of the Alg-Tyr-3APBA-Gel-d and Alg-Tyr-Gel hydrogels was evaluated with a live/dead assay. Human colon cancer HT29 cells were selected as a model and cells were plated on the top of hydrogels and analyzed after 1, 3, and 7 days (Figure S4 and Figure 5). Cells showed a good vitality in both hydrogels over time. However, different cell distribution and aggregation were observed (Figure 5). Indeed, after 7 days, larger clusters were detected in Alg-Tyr-3APBA-Gel-d than in Alg-Tyr-Gel, in which the presence of more isolated and dispersed cells was observed. The presence of the dynamic boronic esters in Alg-Tyr-3APBA-Gel-d generates a dynamic and adaptable microenvironment where the matrix adapts its structure in response to external changes and therefore cells have more freedom to adhere, migrate, and spread [41,42].

## 3. Materials and Methods

### 3.1. Materials

Sodium alginate (W201502-1KG; Sigma-Aldrich, Milan, Italy), type A gelatin from porcine skin (G2500-100G; Sigma-Aldrich, Milan, Italy), tyramine, 3-aminophenylboronic acid, N-hydroxysuccinimide (NHS), MES buffer 0.1 M, phosphate-buffered saline (PBS), peroxidase from horseradish, lyophilized, powder ~150 U/mg (HRP enzyme; 77332-100MG; Sigma-Aldrich, Milan Italy), hydrogen peroxide solution, fetal bovine serum (F7524; Sigma-Aldrich, Italy), penicillin–streptomycin (P0781-100ML; Milan, Sigma-Aldrich, Italy), and McCoy’s 5A modified medium powder for cell culture were provided by Sigma-Aldrich (Milan, Italy). N-(3-dimethylaminopropyl)-N’-ethylcarbodiimide hydrochloride (EDC) was purchased from Tokyo Chemical Industry Co. (TCI, Zwijndrecht, Belgium). Live/Dead Cell Viability Assay kit for 3D and 2D cell culture was obtained from EMD Millipore Corp (Milan, Italy) and HT29 cells (human colorectal adenocarcinoma cell line) were purchased from LGC Standards S.r.L (Milan, Italy).

### 3.2. Functionalization of Sodium Alginate with Tyramine

Two grams (9.260 mmol) of sodium alginate was dissolved in 200 mL of MES buffer 0.1 M pH 5 in a flask at room temperature. Then, 1.775 g (9.260 mmol) of EDC-HCl and 1.073 g (9.260 mmol) of NHS were added and the solution was stirred at room temperature for 40 min. Meanwhile, 1.27 g (9.260 mmol) of tyramine was added to 25 mL MES buffer 0.1 M pH 5 and left under stirring at room temperature. After 40 min, the tyramine solution was added to the solution of sodium alginate. After 40 min, 1.775 g (9.260 mmol) of EDC-HCl and 1.073 g (9.260 mmol) of NHS were added and the reaction mixture was stirred at room temperature overnight. The day after, the solution was dialyzed with a 14 kDa cellulose membrane against NaCl 0.1 M water solution for 24 h and then against distilled water for 48 h. The purified solution was filtered with a 1 µm filter, lyophilized, and stored at −20 °C.

### 3.3. Functionalization of Sodium Alginate with Tyramine and 3-Aminophenylboronic Acid

Two grams (9.260 mmol) of sodium alginate was dissolved in 200 mL of MES buffer 0.1 M pH 5 in a flask at room temperature. Then, 1.775 g (9.260 mmol) of EDC-HCl and 1.073 g (9.260 mmol) of NHS were added and the solution was stirred at room temperature for 40 min. Meanwhile, 1.27 g (9.260 mmol) of tyramine was dissolved in 25 mL MES buffer 0.1 M pH 5 and 0.317 g (2.315 mmol) of 3APBA was dissolved in 50 mL MES buffer 0.1 M pH 5 at room temperature. After 40 min, the solution of tyramine was added to that of sodium alginate. After 40 min, 1.775 g (9.260 mmol) of EDC-HCl and 1.073 g (9.260 mmol) of NHS were added and the reaction mixture was stirred at room temperature for another 40 min. Then, the solution of 3APBA was added and the final solution was stirred at room temperature overnight. The solution was then dialyzed with a 14kDa cellulose membrane against NaCl 0.1 M water solution for 24 h and then against distilled water for 48 h. The product Alg-Tyr-3APBA was filtered with a 1 µm filter, lyophilized, and stored at −20 °C.

### 3.4. Alginate–Gelatin Ink Formulation

Alg-Tyr-3APBA was dissolved in PBS (pH 7.4) in a 2 mL Eppendorf, sonicated, and vortexed at 1200–1600 rpm until complete dissolution (1 h). Gelatin was dissolved in PBS (pH 7.4) at 37 °C. When the polymers were dissolved, gelatin solution was transferred to alginate solution. Then, the enzyme HRP was added to the solution of gelatin and alginate. Finally, H_2_O_2_ was added and vortexed for two minutes at 1200 rpm. Different formulations were tested by changing the molar ratio and the enzymes and H_2_O_2_ amounts as reported in Table 1.

### 3.5. Fourier-Transform Infrared Spectroscopy (FT-IR) and Nuclear Magnetic Resonance (NMR)

FT-IR was performed after freeze drying using a Nicolet iS20, Thermo Scientific; spectra were recorded in transmittance mode in a range of 525–4000 cm^−1^ by setting 32 scans and a resolution of 4 cm^−1^. ^1^H NMR and ^13^C NMR were performed using a Varian Mercury 400 M Hz, dissolving the sample in D_2_O with 0.05% (wt%) TMSP at 40 °C. The data obtained were processed using the MestReNova software.

### 3.6. Swelling Test

The Alg-Tyr-3APBA-Gel-d and Alg-Tyr-Gel hydrogels were formulated in a plastic vial and were weighed after formation. For each hydrogel formulation, three buffer conditions were tested: pH 5.5, 7.4, 8.5 (containing 0.02% w/V NaN_3_). Samples were immersed in 1mL PBS and incubated at 37 °C. To measure the weight variations, PBS was removed from each sample and excess water in the vial was removed and the vials weighed at preconcerted time intervals. The percentage of swelling ratio was calculated with the following formula:

$$Swelling\;Ratio\%=\left[\frac{\left(W_{s}-W_{0}\right)}{W_{0}}\right]∙100$$

where W_s_ indicates the weight of the swollen hydrogel, while W_0_ represents the initial weight of the hydrogel. All the measurements were performed in triplicate.

### 3.7. Rheology

Rheology tests were carried out using an Anton Paar Modular Compact Rheometer MCR 92 equipped with an Anton Paar Measuring Cone Plate CP50-1 (diameter 50 mm, angle 1°). Data were analyzed with RheoCompassTM software. In the amplitude sweep experiment, to determine the LVER, a range of deviation tolerance of ±5% was set.

### 3.8. Self-Healing of Alg-Tyr-3APBA-d

Alg-Tyr-3APBA-d and Alg-Tyr-Gel hydrogels were formulated. To visualize the self-healing properties, control hydrogels were prepared by adding 20 µL of Trypan blue. Briefly, 2.6 mg of Alg-Tyr-3APBAd was dissolved in PBS (pH 7.4) in a 2 mL Eppendorf, sonicated, and vortexed at 1200–1600 rpm until complete dissolution (1 h). Then, 17.3 mg of gelatin was dissolved in PBS (pH 7.4) at 37 °C. When the polymers were dissolved, gelatin solution was transferred to alginate solution. Then, the enzyme HRP (0.5 U/mL) was added to the solution of gelatin and alginate. Finally, H_2_O_2_ (0.5 mM) was added and vortexed for two minutes at 1200 rpm. The formulated hydrogel was first made into a circular shape. After 30 min at 37 °C, the samples were cut by a bistoury and the two halves of different colors were manually reattached. After 10 min at 37 °C, the self-healing test was performed by picking up the sample with tweezers and trying to stretch it to evaluate if the two halves remained attached. The same procedure was performed to produce and compare Alg-Tyr-Gel.

### 3.9. Cell Culture (HT29)

HT29 cells were cultured in McCoy’s 5A complete medium supplemented with 10% fetal bovine serum, 100 mg/mL streptomycin, 100 units/mL penicillin. Cells were grown to 90% confluence, trypsinized, plated in 75 cm^2^ culture dishes at a density of 2 × 10^6^ cells and incubated at 37 °C in a humidified atmosphere containing 5% CO_2_. The medium was changed every two to three days.

### 3.10. Cytotoxicity

The Live/Dead Cell Viability Assay kit (Merk—CBA415) was used to evaluate the cytotoxicity of 3D cell-laden hydrogels. Alg-Tyr-3APBA, Alg-Tyr, and gelatin solutions were sterilized under UV light for 30 min. HRP and H₂O₂ were prepared with autoclaved Milli-Q water to obtain sterility.

Fifty microliters of each hydrogel (formulated as reported in Section 3.2 was plated in triplicate for each time point in a tissue culture-treated 96-well plate with 100 µL of McCoy’s medium with HT29 cells (concentration of 1 × 10^6^ cells/mL) added on the top. At 1, 3, and 7 days, HT29 cells were incubated for 20 min (37 °C, 5% CO_2_) with a solution of red fluorescent ethidium homodimer-1 and green fluorescent calcein-AM. Fluorescence images were collected with a CELENA^®^S, Logos Biosystems. Cell viability was calculated according to the following equation considering the total number of live (green) and dead (red) cells imaged (S4):

*Live/Live* + *Dead* = % *viability*

## 4. Conclusions

In this work, alginate–gelatin bioinspired hydrogels were produced with static and dynamic crosslinking. The proposed method allows the controlled insertion of gelatin without further modifications, or introductions of functional groups, reducing the effort to produce hybrid multifunctional materials and introducing static–dynamic properties able to better mimic the ECM dynamism. From the comparison with different formulations, the static–dynamic Alg-Tyr-3APBA-Gel-d was the most suitable for 3D models’ development since it is transparent, presents a fast gelation time, is cell friendly, and is not cytotoxic. Moreover, this dynamic–static formulation was able to modulate the loss of the inner structure stability for a longer time than the corresponding formulation made with only the covalent crosslinking and showed self-healing properties.

## Figures and Tables

**Table 1:** Formulations of Alg-Tyr-3APBA-Gel and Alg-Tyr-Gel.

Hydrogel	Alginate (mg)	Gelatin (mg)	Tyramine:Tyrosine (Molar Ratio)	HRP (U/mL)	H_2_O_2_ (mM)	Hydrogel% (m/V)	Gelation Time (min)
Alg-Tyr-3APBA-Gel-a	2.6	17.3	2.5:1	1	1	2	1
Alg-Tyr-3APBA-Gel-b	1.1	18.3	1:1	1	1	2	5
Alg-Tyr-3APBA-Gel-c	0.45	18.7	1:2.5	1	1	2	-
Alg-Tyr-3APBA-Gel-d	2.6	17.3	2.5:1	0.5	0.5	2	5
Alg-Tyr-3APBA-Gel-e	1.1	18.3	1:1	0.5	0.5	2	15
Alg-Tyr-Gel	2.6	17.3	2.5:1	0.5	0.5	2	5

## Data Availability

The data presented in this study are available on request from the corresponding author.

## Supplementary Materials

The following supporting information can be downloaded at: https://www.mdpi.com/article/10.3390/molecules28062851/s1, Figure S1: ¹H-NMR of tyramine and 3APBA; Figure S2: ^13^C-NMR of sodium alginate and Alg-Tyr-3APBA; Figure S3: FT-IR spectra of sodium alginate, Alg-Tyr-3APBA, Alg-Tyr; Figure S4: Quantification of living cells detected by live/dead assay at day 1, day 3, and day 7 on hydrogels Alg-Tyr-3APBA-Gel-d (red) and Alg-Tyr-Gel (black). Ref [23,30] are cited in the supplementary materials.

## Sample Availability

Samples of the compounds are available on request from the corresponding author.
