# Supplementary material for: Alginate–Gelatin Self-Healing Hydrogel Produced via Static–Dynamic Crosslinking
Source: Molecules. 2023 Mar 22;28(6):2851. doi: 10.3390/molecules28062851 (PMC10053920; doi:10.3390/molecules28062851)
Supplement: Supplementary file 1 [file molecules-28-02851-s001.zip › molecules-2202797-SI.pdf]

Supporting Information

NMR. In Figure S1 were reported the ^{1}H-NMR spectra of Tyramine and 3-APBA. The peaks related to the aromatic protons of 3-APBA were identified in a range between δ=7-7,4 ppm [1] while the aromatic protons in orto position respect to the hydroxyl group peaks of Tyramine were visible δ=6,8 and the protons in meta position at δ=7,1 ppm [2]. Moreover, the protons of -CH_2 chain of Tyramine are visible between δ=2,7-3,0 ppm.

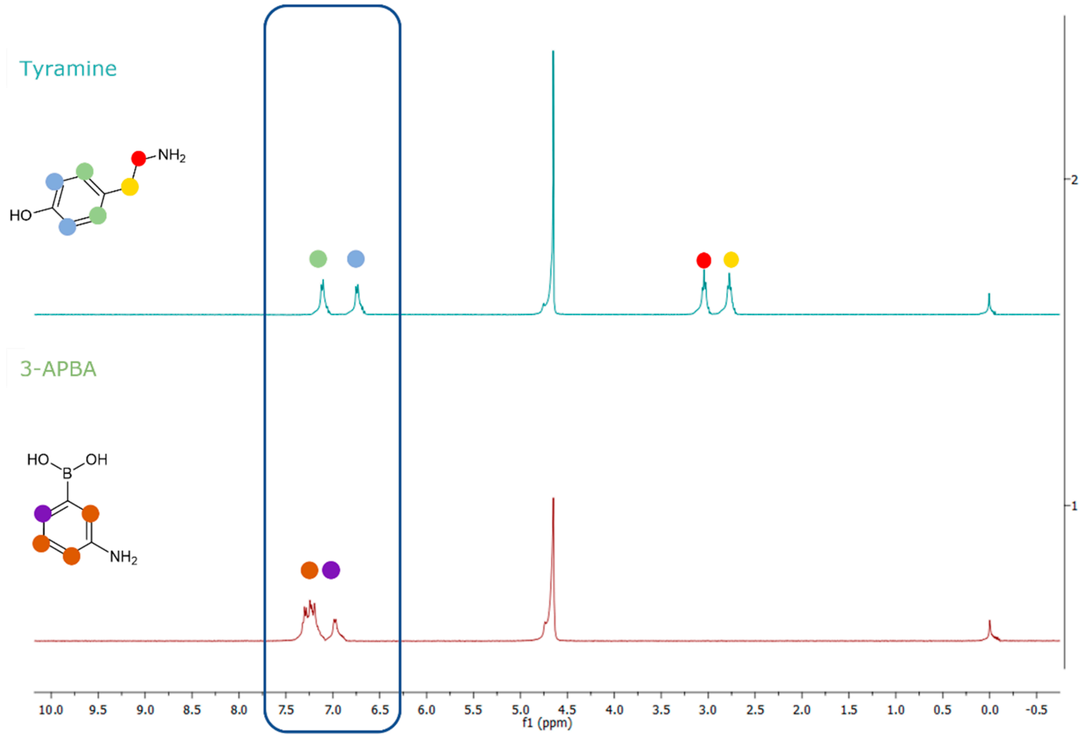

Figure S1. ^{1}H-NMR of Tyramine and 3-APBA.

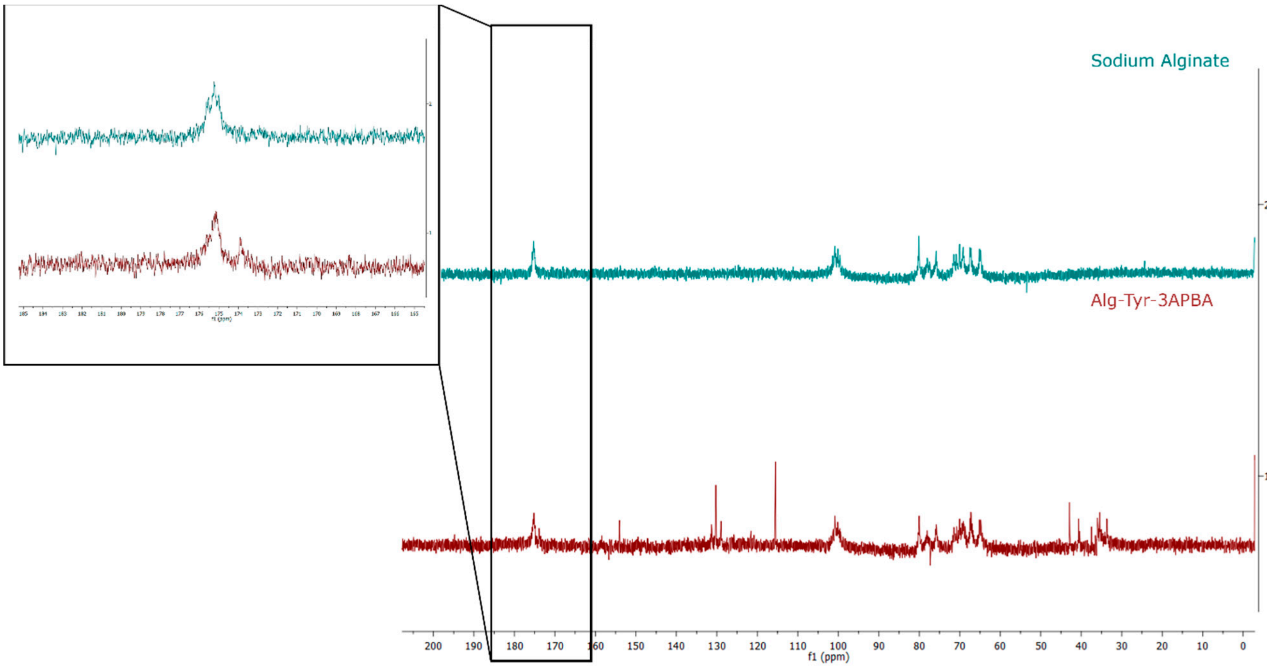

Figure S2. ^{13}C-NMR of Sodium Alginate and Alg-Tyr-3-APBA.

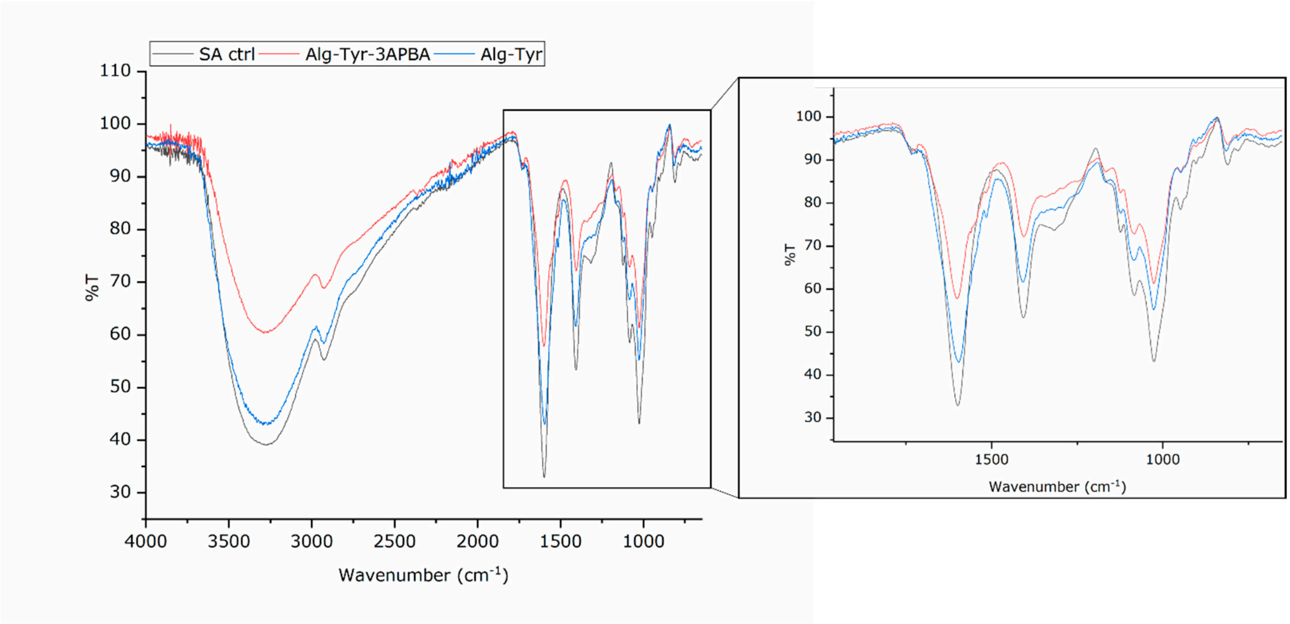

Figure S3. FT-IR spectra of sodium alginate, Alg-Tyr-3APBA, Alg-Tyr

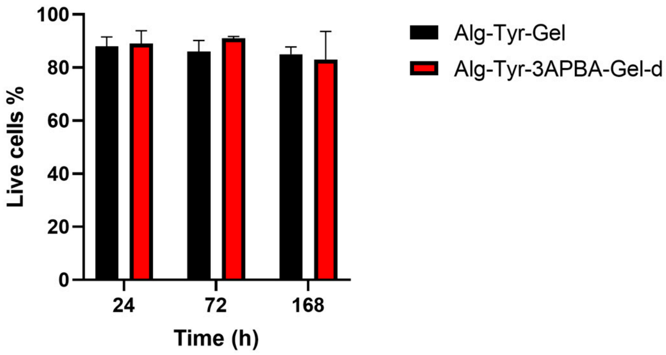

Figure S4. Quantification of living cells detected by Live-Dead assay at day 1, day 3 and day 7 on hydrogels Alg-Tyr-3APBA-Gel-d (red) and Alg-Tyr-Gel (black).

Reference

1. Hong, S.H.; Kim, S.; Park, J.P.; Shin, M.; Kim, K.; Ryu, J.H.; Lee, H. Dynamic Bonds between Boronic Acid and Alginate: Hydrogels with Stretchable, Self-Healing, Stimuli-Responsive, Remoldable, and Adhesive Properties. *Biomacromolecules* **2018**, *19*, 2053–2061, doi:10.1021/ACS.BIOMAC.8B00144.

2. Schulz, A.; Gepp, M.M.; Stracke, F.; von Briesen, H.; Neubauer, J.C.; Zimmermann, H. Tyramine-Conjugated Alginate Hydrogels as a Platform for Bioactive Scaffolds. *J. Biomed. Mater. Res. A* **2019**, *107*, 114–121, doi:10.1002/JBM.A.36538.
